# Plant Growth Promotion and Induction of Systemic Tolerance to Drought and Salt Stress of Plants by Quorum Sensing Auto-Inducers of the *N*-acyl-homoserine Lactone Type: Recent Developments

**DOI:** 10.3389/fpls.2021.683546

**Published:** 2021-05-31

**Authors:** Anton Hartmann, Sophia Klink, Michael Rothballer

**Affiliations:** ^1^Microbe-Host Interactions, Faculty of Biology, Ludwig-Maximilians-Universität München, Planegg, Germany; ^2^Institute of Network Biology, Helmholtz Zentrum München, Neuherberg, Germany

**Keywords:** *N*-acyl-homoserine lactones, priming, induced systemic tolerance, ACC deaminase, auxins, osmolytes

## Introduction

During the evolution of land plants, plant-associated microbiomes co-evolved and were integrated into prokaryote-eukaryote holobionts. Functions of these metagenomically organized plants are based on the gene expression of all holobiont participants. The constitution of the holobiont is dependent on specific signaling and perception events between the microbial and plant partners. Microbially produced plant hormones and a diversity of other metabolites have key roles in the process of interkingdom interaction of organisms. In association/symbiosis with plant beneficial bacteria, the ability of plants to cope with abiotic and biotic stresses is enhanced. As example for systemic effects reducing biotic stress, AHL-producing *Serratia liquefaciens* MG1 conferred biocontrol of the phytopathogenic fungus *Alternaria alternata* in tomato plants (Schuhegger et al., 2006). The down-regulation of the stress hormone ethylene by degradation of the precursor 1-aminocyclopropane-1-carboxylate (ACC) with ACC deaminases (Glick, 2014) has a key role in the beneficial interaction of bacteria with plants under abiotic stress conditions. The ability of degrading the ethylene precursor ACC is frequently found in bacteria isolated from the rhizosphere of salt-tolerant plants (Jha et al., 2012; Suarez et al., 2015; Hrynkiewicz et al., 2019). Other mechanisms for the mitigation of salinity and drought stress in plants, like the involvement of IAA by rhizobacteria (Egamberdieva et al., 2017), antioxidants, extracellular polymeric substances (EPS) or volatile organics were recently reviewed by Kumar et al. (2020). However, the induction of systemic tolerance to abiotic stress conditions by the quorum sensing auto-inducers of *N*-acyl-homoserine lactone-type (AHL) or AHL-producing rhizobacteria was not mentioned in this recent review and is generally not much recognized yet.

Since more than two decades, a very sensitive and effective communication system within bacterial populations is known, based on auto-inducers. In Gram-negative bacteria, molecules of the *N*-acyl-homoserine lactone type with hydrophilic (short) and hydrophobic (long) CH-chain are produced by *luxI-*type genes (Hartmann and Schikora, 2012; Rothballer et al., 2018). These small molecules are synthesized by the bacteria themselves but also by others in their vicinity depending on environmental conditions in the bacterial habitat. Their perception and subsequent concentration dependent transcriptional regulation mediated by *luxR*-type receptors allow to efficiently adapt gene expression to environmental changes. This makes the auto-inducer signaling an essential and evolutionary relevant tool for the efficiency of bacterial behavior (Hense and Schuster, 2015). Interkingdom signaling of bacteria with host plants based on AHL auto-inducers was demonstrated in many Gram-negative rhizobacteria as reviewed by Schikora et al. (2016). The most recently observed role of AHLs in the improvement of abiotic stress tolerance of some plants deserves to be widened for more different crops to be finally applied in practical agriculture.

## Roles of Phytohormones and Osmolytes in Salt and Drought Tolerance of Plants

Plant hormones play an essential role in the regulation of responses at environmental stress situations such as drought or high salinity. It has been reviewed in detail, which different mechanisms are active in a wide number of diverse plant growth promoting rhizobacteria (PGPR) conferring drought and salt tolerance to crop plants (Vurukonda et al., 2016). In all living organisms the key physiological basis of salt and drought tolerance is the accumulation of so-called osmolytes. Important osmolytes are certain amino acids (especially proline), amines (glycine betaine or ectoine) and sugars, which have specific physicochemical properties to protect cellular protein structures and balance high osmotic pressure. Oxygen stress is also accompanying abiotic and biotic stress situations. Therefore, the oxygen radical scavenging enzymes are also very important in these situations. For example, the oxygen radical scavenging enzyme superoxide dismutase of *Gluconacetobacter diazotrophicus* PAL5 is obligatory for endophytic colonization of rice (Alqueres et al., 2013). Concerning the hormonal drought-stress responses in plants, abscisic acid (ABA) and other phytohormones, such as jasmonates and ethylene are involved. In addition, the auxin indole-3-acetic acid (IAA) in an optimal ratio to other phytohormones plays an integral part in plant adaptation to water stress. It is therefore of particular importance under abiotic stress conditions that root associated bacteria themselves produce and excrete auxins and other phytohormones or influence the phytohormone balance supporting plants which have too low auxin levels due to salt inhibition. The use of salt-tolerant phytohormone producing bacteria in combination with high osmolyte containing seaweeds is widely distributed and successfully applied to support crop growth in salt affected soils as reviewed by Nabti et al. (2015). Another approach to solve the lack of IAA in plants under salt stress is the inoculation with IAA-overproducing *Ensifer meliloti* conferring drought tolerance to *Medicago sativa* (Defez et al., 2017b). Inoculation with IAA-overproducing strains of endophytic *Burkholderia cepacia* strain RRE25 caused stimulated root development and improved nutrient use efficiency (especially of phosphate uptake) leading to increased plant growth (Singh et al., 2013). In endophytic diazotrophic bacteria *Enterobacter cloacae* RCA25 and *Klebsiella variicola* RCA26 overproduction of IAA upregulated nitrogen fixation in bacterial cultures and inoculated rice plants (Defez et al., 2017a). *Azospirillum brasilense* Cd mutants which overproduce IAA could be selected using the L-tryptophan toxic antimetabolite 5-fluor-tryptophan (Hartmann et al., 1983), leading to plants with increased root system at low nitrogen supply. Furthermore, using the proline antimetabolite 3-dehydroproline (DHP), *A. brasilense* Sp7 mutants with increased osmotolerance have been isolated (Hartmann et al., 1992). Concerning proline-overproducing strains, many salt-tolerant bacteria are already DHP-resistant. The screening for DHP-resistance was not yet systematically applied to yield strains with improved salt and drought tolerance.

## Quorum Sensing Autoinducer *N*-Acyl-Homoserine Lactones and AHL-Producing Rhizobacteria Stimulate Plant Growth Under Normal and Abiotic Stress Conditions

The influence of AHLs on plant performance is dependent on the molecular structure of the AHL-signal. Treatment with water-soluble C4- and C6-homoserine lactones (HSL) changes in the phytohormone balance occurred in roots and shoots of *Arabidopsis thaliana* (von Rad et al., 2008). It was found that the presence of GCR1/GPA1 genes in the plant host mediates the stimulation of root growth (Liu et al., 2012). In barley plants, AHL-molecules applied in axenic model experiments with seedlings initiated a nitrous oxide (NO)-burst and Ca^2+^-fluxes in root cells as well as morphological changes in roots (Rankl et al., 2016). As shown in *A. thaliana*, calmodulin receptors were involved in primary root elongation stimulated by 3-oxo-C6 HSL (Zhao et al., 2015). Furthermore, it was shown that ATMYB44 was involved in the enhanced elongation of primary roots (Zhao et al., 2016). Using several independent experimental approaches, it could be clearly proven that water-soluble AHLs are taken up by plant roots through the vascular system into the shoot by an energy-dependent process. However, this is only possible in plants, like *Arabidopsis thaliana*, wheat, or barley, which are devoid of AHL-degrading enzymes, like lactonases. In plant shoots, hydrophilic C4-, C6-, and C8-HSLs influence the activities of enzymes with e.g., anti-oxidative capacity or xenobiotic phase II detoxifying enzymes improving stress tolerance (Goetz-Roesch et al., 2015).

Water-insoluble AHLs with long CH-chains (e.g., C12- and C14-HSL) are also effective in modifying plant performance (Schenk and Schikora, 2015). Several lines of evidences point to the involvement of a specific receptor for water-insoluble AHLs in plant cells and the activation of a specific signaling cascade. In barley it was demonstrated that the ability of AHL-priming of systemic resistance is a genetically determined property (Wehner et al., 2019). Salicylic acid (SA) and oxylipin 12-oxo-phytodienoic acid (*cis*-OPDA) are involved in the systemic response and priming (Schenk and Schikora, 2015; Shrestha et al., 2020). In addition, the activation of MAP-kinases MPK3 and MPK6 were enhanced and prolonged, along with the upregulation of defense-related transcription factors *WRKY22* and *WRKY29*, glutathione-S-transferase *GST6*-gene and heat shock protein *Hsp60*-gene in *Arabidopsis* by a mixture of short- and long CH-chain AHLs (Shrestha et al., 2019). AHL-producing *Burkholderia graminis* M12 and M14 strains could induce tolerance to salt stress in wild type and genetically engineered tomato plants (Barriuso et al., 2008). It was recently shown by Zhao et al. (2020) that AHL-enhanced tolerance to salt stress in *Arabidopsis thaliana* and wheat was linked to ABA-dependent and ABA-independent signaling pathways and also the Salt Overly Sensitive (SOS) pathway. 3-oxo-C6-HSL treated plants had an increased proline and chlorophyll content, while lipid peroxidation, as measured by tissue content of malondialdehyde, and the Na^+^/K^+^ ratio was decreased. It was clearly demonstrated that inoculation with AHL-producing plant beneficial Gram-negative bacteria like *Gluconacetobacter diazotrophicus* PAL5 supports plant growth under adverse conditions. For example, *G. diazotrophicus* mitigates drought stress in *Oryza sativa* (Filgueiras et al., 2020), resulting in higher biomass production, higher levels of gas exchange and osmoprotecting solutes (proline and glycine betaine) in shoots and roots under drought conditions. Under reduced water availability malondialdehyde accumulation, a product of lipid peroxidation, increased drastically as result of cell damages, but in PAL5-inoculated plants the accumulation of malondialdehyde was much reduced. In addition, the anti-oxidant enzymes superoxide dismutase (*sodA*), glutathione reductase (*gor*), catalase (*katE*), pyrrolinbe-5-carboxylate reductase (*P5CR*) and betaine aldehyde dehydrogenase (*BADH*) were stimulated by inoculation with *G. diazotrophicus* PAL5 under increased water stress. Interestingly, this “induced systemic tolerance” at reduced water availabilities was accompanied by an increased transcription of both pathogenesis related PR-1 and PR-10 genes, which are known to function in the salicylic acid (SA) and jasmonic acid (JA) pathways. Furthermore, in *G. diazotrophicus* PAL5 exposed to reduced water availability, AHL synthase gene expression increased dramatically, which is linked to a stimulation of *N*-acyl-homoserine lactone production. Thus, AHLs are probably the key bacterial signals behind the induced systemic tolerance response in red rice, reported by Filgueiras et al. (2020).

## AHL-Devoid Mutants Are Impaired in Beneficial Plant Effects

The central role of AHLs in the plant beneficial effects was proven several times with mutants lacking the *luxI*- or *luxR* gene homologue or in AHL-depleted transconjugants of PGPR. *Rhizobium radiobacter* F4 has been first demonstrated as endofungal bacterium of the plant growth promoting fungus *Serendipita indica* (syn. *Piriformospora indica*) (Sharma et al., 2008; Varma et al., 2012). However, even without the fungus, *R. radiobacter* F4 (RrF4) proved to have very similar plant beneficial effects as *S. indica*, like improved abiotic and biotic stress tolerance (Glaeser et al., 2015). Recently, it was demonstrated that *R. radiobacter* F4 produced a spectrum of short and medium CH-chain AHL molecules (Alabid et al., 2020). *R. radiobacter* transconjugants F4NM13 with a lactonase-producing plasmid, which are devoid of AHLs, showed only reduced colonization abilities of roots of *Arabidopsis thaliana* and barley and only marginal plant beneficial effects. In the PGPR *Acidovorax radicis* N35 (Li, 2010), producing 3-OH-C10-HSL, *araI*-deleted mutants were less effective in root colonization in competition with the wild type. While the AHL-producing wild type induced a plant expression profile of stimulation and some priming, the AHL-deleted mutant caused increased expression of defense responses like flavonoid biosynthesis (Han et al., 2016). Thus, in *A. radicis*, AHL-production could influence the perception by a host plant. This was also indicated by *Arabidopsis thaliana* and barley inoculation with the AHL-negative tranconjugant *Rhizobium radiobacter* F4NM13 (Alabid et al., 2020), but more studies are necessary to further document this important issue.

## Possible Roles of *LuxR*-Solos in Plant Beneficial Effects

In the frequently applied *Azospirillum brasilense* strains Ab-V5, Sp245, Sp7, or Az39 and also in other *Azospirillum* species strains (Gualpa et al., 2019) *luxR*-solo or *luxR*-orphan genes were found, i.e., *luxR*-homologous receptors without the presence of a *luxI*-type AHL-synthase. It has been shown by Fukami et al. (2018) that the addition of 3-oxo-C6-HSL to *luxR*-solo *A. brasilense* Ab-V5 caused a significant stimulation of plant colonization traits, demonstrating that external available AHLs were perceived and resulted in a positive response of stimulated bacteria-plant interaction. It has been proposed by Patel et al. (2013) that bacterial *luxR*-solos have evolved to respond to AHL-related or even rather unrelated signals from plants. This evolution of the *luxI-/luxR*-type quorum sensing regulon may be regarded as the basis of a novel signal/receptor couple for plant-bacteria communication (Schaefer et al., 2013, 2016). According to Gualpa et al. (2019), quorum quenching activities were prevalent in *A. brasilense* Az39, which also lacks a *luxI*-type AHL-biosynthesis gene. It may be possible, that *A. brasilense* Az39 has already gone steps forward in the evolution of the QS system and specialized on the degradation of AHLs. This may provide the bacterium an advantage to neutralize competing AHL-producing bacteria in the rhizosphere and in parallel facilitates receiving communication signals from the plant through *luxR*-solo receptors.

## Future Perspectives

**FIGURE 1:**
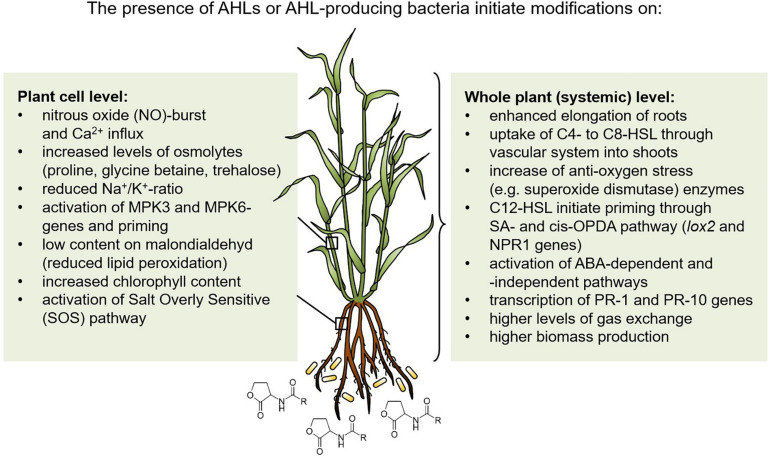
Induced systemic tolerance against drought and salt stress in *N*-acyl-homoserine lactone-treated plants. MPK, Mitogen-associated protein kinase; SA, Salicylic acid; *cis*-OPDA, 12-oxo-phytodienoic acid; PR-1 and PR-10, Pathogenesis related proteins; ABA, Abscisic acid; lox, Lipoxygenase; NPR1, Nonexpressor of pathogenesis-related genes 1.

A high diversity of rhizobacteria support plants in environmental stress conditions, especially under salt and drought stress using different mechanisms (Kumar et al., 2020). In addition, QS signals of the *N*-acyl-homoserine lactone-type from Gram-negative bacteria stimulate plant development and prime systemic tolerance to environmental stress in crop plants. This stimulation occurs at cellular as well as whole plant level, as summarized in Figure 1. Thus, it should be a desirable goal to use AHL-producing bacteria or their AHL-signals as well as AHL quorum sensing related activities to stimulate plant tolerance to abiotic stress. In this context, synthetic biology would be a helpful approach (Stephens and Bentley, 2020). The entire network of interactions of the plant microbiome with host plants under different biotic and abiotic stress situations needs to be further developed also in a systems biology approach (Rodriguez et al., 2019). Besides the stimulatory interaction of AHL-producing bacteria with plants, C4- and C6-HSL molecules as foliar spray or seed treatment should be applied to improve plant performance, yields and resistance to biotic and abiotic stresses as was successfully demonstrated by Moshynets et al. (2019). Furthermore, a nanocomposite fertilizer using magnetic carbon nanofibers coupled with C4-HSL stimulated seed germination and growth of crops with enhanced resistance to oxidative and high salinity stresses (Gupta et al., 2019). Thus, the application of a synthetic AHL concentrate for bacteria-plant stimulation started already quite promising and AHL-QS research should be emphasized in the future involving more different crop plants. However, it should be kept in mind that not all cultivars are susceptible for AHL-perception (Shrestha et al., 2019). More basic understanding of the underlying mechanisms should further support successful field applications to modify and improve crop growth in abiotic and biotic challenging conditions.

## Author Contributions

AH designed the structure and content of the manuscript. SK wrote part of the manuscript, corrected the final manuscript, and contributed the figure. MR wrote part of the manuscript and corrected the final manuscript. All authors contributed to the article and approved the submitted version.

## Conflict of Interest

The authors declare that the research was conducted in the absence of any commercial or financial relationships that could be construed as a potential conflict of interest.
